# Prediction of Conversion From Amnestic Mild Cognitive Impairment to Alzheimer's Disease Based on the Brain Structural Connectome

**DOI:** 10.3389/fneur.2018.01178

**Published:** 2019-01-10

**Authors:** Yu Sun, Qiuhui Bi, Xiaoni Wang, Xiaochen Hu, Huijie Li, Xiaobo Li, Ting Ma, Jie Lu, Piu Chan, Ni Shu, Ying Han

**Affiliations:** ^1^Department of Neurology, XuanWu Hospital of Capital Medical University, Beijing, China; ^2^State Key Laboratory of Cognitive Neuroscience and Learning & IDG/McGovern Institute for Brain Research, Beijing Normal University, Beijing, China; ^3^Center for Collaboration and Innovation in Brain and Learning Sciences, Beijing Normal University, Beijing, China; ^4^Beijing Key Laboratory of Brain Imaging and Connectomics, Beijing Normal University, Beijing, China; ^5^Department of Psychiatry and Psychotherapy, Medical Faculty, University of Cologne, Cologne, Germany; ^6^Department of Psychology, University of Chinese Academy of Sciences, Beijing, China; ^7^CAS Key Laboratory of Behavioral Science, Institute of Psychology, Beijing, China; ^8^Department of Biomedical Engineering, New Jersey Institute of Technology, Newark, NJ, United States; ^9^Department of Electronic and Information Engineering, Harbin Institute of Technology Shenzhen Graduate School, Shenzhen, China; ^10^Department of Radiology, XuanWu Hospital of Capital Medical University, Beijing, China; ^11^Beijing Institute of Geriatrics, XuanWu Hospital of Capital Medical University, Beijing, China; ^12^National Clinical Research Center for Geriatric Disorders, Beijing, China; ^13^Center of Alzheimer's Disease, Beijing Institute for Brain Disorders, Beijing, China

**Keywords:** brain network, conversion, diffusion tensor imaging, graph theory, mild cognitive impairment, machine learning

## Abstract

**Background:** Early prediction of disease progression in patients with amnestic mild cognitive impairment (aMCI) is important for early diagnosis and intervention of Alzheimer's disease (AD). Previous brain network studies have suggested topological disruptions of the brain connectome in aMCI patients. However, whether brain connectome markers at baseline can predict longitudinal conversion from aMCI to AD remains largely unknown.

**Methods:** In this study, 52 patients with aMCI and 26 demographically matched healthy controls from a longitudinal cohort were evaluated. During 2 years of follow-up, 26 patients with aMCI were retrospectively classified as aMCI converters and 26 patients remained stable as aMCI non-converters based on whether they were subsequently diagnosed with AD. For each participant, diffusion tensor imaging at baseline and deterministic tractography were used to map the whole-brain white matter structural connectome. Graph theoretical analysis was applied to investigate the convergent and divergent connectivity patterns of structural connectome between aMCI converters and non-converters.

**Results:** Disrupted topological organization of the brain structural connectome were identified in both aMCI converters and non-converters. More severe disruptions of structural connectivity in aMCI converters compared with non-converters were found, especially in the default-mode network regions and connections. Finally, a support vector machine-based classification demonstrated the good discriminative ability of structural connectivity in differentiating aMCI patients from controls with an accuracy of 98%, and in discriminating converters from non-converters with an accuracy of 81%.

**Conclusion:** Our study provides potential structural connectome/connectivity-based biomarkers for predicting disease progression in aMCI, which is important for the early diagnosis of AD.

## Introduction

Mild cognitive impairment (MCI) is generally associated with a higher risk of dementia and is considered as an intermediate stage between normal aging and Alzheimer's disease (AD) (1–3). A prospective population-based study in elders showed that the incidence of dementia was highest for patients with amnestic MCI (aMCI) (4). However, not all patients with aMCI progress to dementia (5). Early prediction and identification of individuals with aMCI who are at high risk for conversion to AD aids timely detection of dementia, which is essential for early intervention strategies.

Previous studies have shown the potential of imaging markers to predict conversion from MCI to AD dementia. Among multiple neuroimaging modalities, MRI has attracted significant interests due to its completely non-invasive nature, high availability in mild symptomatic patients and high spatial resolution. Structural MRI biomarkers such as gray matter atrophy in the medial temporal lobe (6) and hippocampal/entorhinal cortex (7) have been identified as efficacious AD-specific biomarkers for the early diagnosis and prediction of disease progression. With diffusion MRI techniques, promising markers of microstructural white matter (WM) damage in AD and MCI patients have been proposed (5, 8, 9). Specifically, regional diffusion metrics of limbic WM in the fornix, posterior cingulum, and parahippocampal gyrus have shown better performance than volumetric measurements of gray matter in predicting MCI conversion (10–14).

However, compared with local or regional imaging markers, the network model has provided a new perspective to investigate the neuropathological progression of AD from a system level (15–18). The whole-brain WM structural network at macroscopic level can be constructed with diffusion MRI and tractography approaches. The topological organization of brain network can be further characterized with graph theoretical analysis (for reviews, see (19, 20). Several non-trivial topological properties, such as small-worldness, modular structure, and rich-club organization of WM networks have been consistently demonstrated in healthy population (21, 22). For AD and aMCI, previous WM network studies have suggested that AD patients exhibit decreased topological efficiency than healthy controls, which is associated with cognitive decline (23, 24). Similarly, our previous work has also found decreased network efficiency in patients with aMCI (25–27) and in those at an earlier stage (28). Importantly, hub regions are preferentially disrupted in AD and aMCI patients, especially the default mode network (DMN) regions, which concentrated most of the pathology of Aβ deposition (29–32). These findings suggest potential, sensitive connectome-based markers for the early detection of structural alterations due to pathological or/and neurodegenerative processes in the early stages of AD. Recently, machine learning, deep learning and complex brain networks have been recently applied to the early diagnosis of neurodegenerative diseases with interesting results (33–36). Specifically, functional MRI network studies have found more severe disruptions in MCI converters, which may distinguish converters from non-converters with high accuracy (37–39). Structural MRI studies have also found topological differences of brain connectome between the two groups (40–42). However, whether the structural brain connectome can provide sensitive markers to predict longitudinal conversion from aMCI to AD has remained largely unknown.

Thus, in our study, we focused on aMCI patients who progressed to probable AD in 2 years after their baseline scan (referred to as “aMCI converters”) and compared them with aMCI patients who were clinically stable (i.e., did not develop AD) during 2 years follow-up (referred to as “aMCI non-converters”). Diffusion MRI tractography and graph theory approaches were performed to investigate baseline differences in the topological organization of the WM structural networks between aMCI converters and non-converters. We sought to determine (1) whether the WM networks would show progressive alterations in aMCI converters compared with non-converters, (2) how network disruptions would predict disease progression in aMCI patients, and (3) the potential utility of brain structural connectome for individual prediction and diagnosis in the early stage of AD.

## Materials and Methods

### Participants

This retrospective study involved 78 elderly subjects, including 52 aMCI patients, who were recruited from the Memory Clinic of the Neurology Department, XuanWu Hospital, Capital Medical University, Beijing, China and 26 demographically matched healthy controls (HCs) who were recruited from local communities. The inclusive criteria of aMCI patients were proposed by Petersen (43, 44) and described as follows: (1) definite complaints of memory declined, preferably confirmed by an informant; (2) objective cognitive performances in single or multiple domains including memory documented by neuropsychological tests scores were below or equal to 1.5 SD of age- and education-adjusted norms; (3) a Clinical Dementia Rating (CDR) score of 0.5; (4) preservation of independence in activities of daily living; and (5) not sufficient to meet the criteria for dementia based on DSM-IV-R (Diagnostic and Statistical Manual of Mental Disorders, 4th edition, revised). Subjects who had no complaints of cognition and normal objective cognitive performances as well as a CDR score of 0 were referred as HCs. The exclusive criteria of participants were as follows: (1) a history of stroke, traumatic brain injury, neurological/psychiatric diseases, and other central nervous system diseases that may lead to cognitive impairment; (2) major depression (Hamilton Depression Rating Scale score >24 points); (3) other systemic diseases including thyroid dysfunction, syphilis, severe anemia, or HIV that may cause cognitive impairment; (4) addictions or treatments that would influence cognitive ability; (5) vessel disease included cortical and/or subcortical infarcts, or WM hyperintensity and lesions; (6) severe visual or auditory disabilities.

These participants were selected from a larger cohort (*n* = 205) and consisted of those who had completed MRI scanning at baseline and undergone a 2 years longitudinal follow-up at least once. During follow-up, patients with aMCI were reclassified as aMCI converters (aMCI-c) or aMCI non-converters (aMCI-nc) based on whether they were subsequently diagnosed with dementia. The diagnosis of dementia was triggered by a change in the CDR score from 0.5 to 1.0 and confirmed by neuropsychological tests and physician evaluations. This study included 26 aMCI-c who converted to AD within 2 years and 26 demographically matched aMCI-nc who remained stable during the follow-up.

All participants underwent regular neuropsychological assessments, including the Mini-Mental State Examination (MMSE) (45), Montreal Cognitive Assessment (MoCA) (46), Auditory Verbal Learning Test (AVLT), CDR (47), Hamilton Depression Rating Scale (48), and Activities of Daily Living scale. The study was registered on ClinicalTrials.gov (Identifier: NCT02225964) and study protocol was approved by XuanWu Hospital of Capital Medical University institutional review board, and all participants completed a written informed consent process before any study procedures. Table 1 summarized the main demographic and clinical information of all participants.

**Table 1:** Demographics and neuropsychological testing.

	**aMCI-c (*n* = 26)**	**aMCI-nc (*n* = 26)**	**HC (*n* = 26)**	***F* value**	***P*-value**
Age (years)	67.7 ± 8.1 (50–78)	67.7 ± 8.3 (50–78)	67.8 ± 8.0 (50–78)	0.01	0.99^*^
Gender(M/F)	12/14	14/12	13/13	–	0.86^#^
Education (years)	10.1 ± 5.0 (0–20)	9.4 ± 5.0 (0–18)	11.2 ± 5.4 (0–18)	0.63	0.54^*^
MMSE	23.1 ± 2.9 (17–28)	25.3 ± 3.5 (18–30)	28.0 ± 2.3 (20–30)	20.29	<0.001^*^^a^^b^^c^
MoCA	17.6 ± 3.1 (10–23)	20.6 ± 4.0 (14–26)	26.5 ± 1.5 (25–30)	58.54	<0.001^*^^a^^b^^c^
AVLT-Immediate Recall	4.8 ± 1.2 (2.7–7.3)	5.8 ± 1.7 (3.3–10.0)	9.3 ± 2.0 (2.7–14.7)	53.07	<0.001^*^^b^^c^
AVLT-Delayed Recall	2.7 ± 2.1 (0–6)	3.5 ± 3.1 (0–11)	10.1 ± 2.8 (4–15)	59.08	<0.001^*^^b^^c^
AVLT-Recognition	6.5 ± 3.9 (−3–13)	7.5 ± 3.7 (0–14)	12.2 ± 2.3 (5–15)	20.22	<0.001^*^^b^^c^

*Values are represented as the mean ± SD (range). All of the scores are raw values*.

*HC, healthy control; aMCI, amnestic mild cognitive impairment; aMCI-c, aMCI converters; aMCI-nc, aMCI non-converters; MMSE, Mini-Mental State Examination (Chinese Version); MoCA, Montreal Cognitive Assessment (Beijing Version); AVLT, Auditory Verbal Learning Test*.

* *The P-values were obtained using one-way analysis of variance (ANOVA). Post-hoc pairwise comparisons were performed using a t-test. P < 0.05 was considered significant*.

# *The P-values were obtained using the Kruskal-Wallis one-way ANOVA*.

a *post-hoc paired comparisons showed a significant group difference between aMCI-c vs. aMCI-nc*.

b *post-hoc paired comparisons showed a significant group difference between aMCI-c vs. HC*.

c *post-hoc paired comparisons showed a significant group difference between aMCI-nc vs. HC*.

### Data Acquisition

All participants were scanned using a Siemens Trio 3.0 T MRI scanner at XuanWu Hospital of Capital Medical University. Participants lay still with their heads fixed by straps and foam to minimize movement. The T1-weighted images were acquired using a magnetization prepared rapid gradient echo (MPRAGE) sequence with the following parameters: repetition time (TR) = 1,900 ms; echo time (TE) = 2.2 ms; flip angle = 9°; acquisition matrix = 256 × 224; field of view (FOV) = 256 × 224 mm^2^; slice thickness = 1 mm; no gap; 176 sagittal slices; and average = 1. The diffusion tensor imaging (DTI) data were acquired using a single-shot EPI sequence with the following parameters: TR = 11,000 ms; TE = 98 ms; flip angle = 90°; acquisition matrix = 128 × 116; FOV = 256 × 232 mm^2^; slice thickness = 2 mm; no gap; 60 axial slices; and average = 3. Thirty non-linear diffusion weighting directions with b = 1,000 s/mm^2^ and one b0 image were obtained. All images were reviewed and the leukoencephalopathy and vascular comorbidity was evaluated by an experienced neuroradiologist.

### Data Preprocessing

First, the DTI data was preprocessed to remove the effect of eddy current distortion and motion artifact by applying an affine alignment of the diffusion-weighted images to the reference b0 image. Then the transformation was applied to reorient the b-matrix. Second, the diffusion tensor was calculated and diagonalized to obtain 3 eigenvalues (λ_1_, λ_2_, λ_3_) and their corresponding eigenvectors. Finally, the FA image was calculated. The preprocessing procedure was performed with the FMRIB Diffusion Toolbox (FDT) in FSL (version 5.0, http://fsl.fmrib.ox.ac.uk/fsl/fslwiki/FDT).

### Brain Network Construction

For each participant, the individual WM structural network was constructed with the following procedures.

#### Network Node Definition

**Figure 1:**
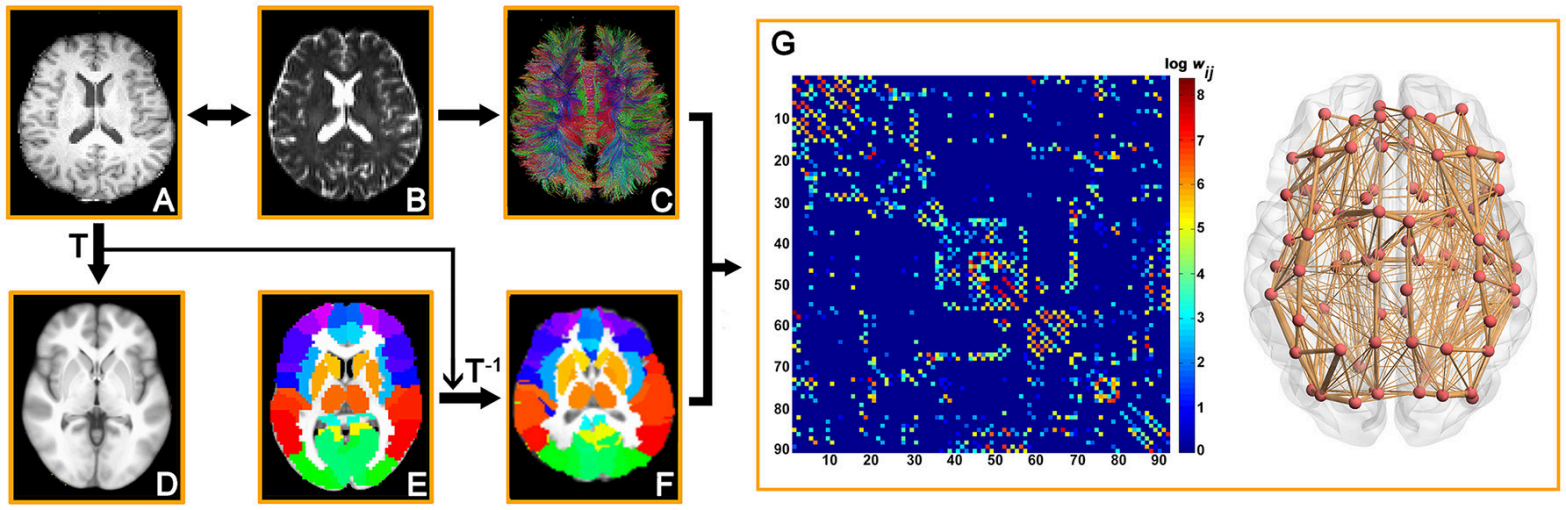
Flowchart for construction of the WM structural network by DTI. (1) Coregistration from an individual T1-weighted image **(A)** to a DTI b0 image **(B)**. (2) Nonlinear registration from the T1-weighted image in the native DTI space to the ICBM152 T1 template in the MNI space **(D)**. (3) Application of the inverse transformation (T^−1^) to the AAL atlas in the MNI space **(E)**, which results in individual-specific parcellation in the native DTI space **(F)**. (4) The reconstruction of the whole-brain WM fibers **(C)** was performed using deterministic tractography in Diffusion Toolkit. (5) The weighted networks of each subject **(G)** were created by computing the number of the streamlines that connected each pair of brain regions. The connection matrix and 3D representation (axial view) of the WM structural network of a representative healthy subject are shown in the right panel. The nodes are located according to their centroid stereotaxic coordinates and the edges are sized according to their connection weights.

To define the network node, we used the Automated Anatomical Labeling (AAL) atlas to parcellate the brain into 90 regions (49). Briefly, T1-weighted image was coregistered to the b0 image in DTI space. Then the transformed T1 images were normalized to the ICBM152 T1 template in the Montreal Neurological Institute (MNI) space. Finally, inverse transformations were applied to AAL atlas to obtain an individual parcellation of 90 ROIs (45 for each hemisphere, Table S1), each representing a node of the network (Figure 1). All procedures were performed using the SPM8 software (https://www.fil.ion.ucl.ac.uk/spm/software/SPM8/).

#### WM Tractography

Deterministic tractography was performed to reconstruct the whole-brain fiber streamlines, by seeding each voxel with an FA >0.2. The tractography was terminated if it reached a voxel with an FA <0.2 or turned an angle >45 degrees (50). The tractography was performed using Diffusion Toolkit (http://www.trackvis.org/dtk/) based on the “fiber assignment by continuous tracking” method (50).

#### Network Edge Definition

Between each pair of ROIs, the weight of the edge was defined as the number of fiber streamlines (FN) with two end points located in these two regions. Therefore, an FN-weighted 90 × 90 structural connectivity (SC) network was constructed for each participant (Figure 1).

### Network Analysis

#### Small-World Properties

Several graph metrics were calculated to quantify the topological organization of WM structural networks, including network strength (S_p_), global efficiency (E_glob_), local efficiency (E_loc_), shortest path length (L_p_), clustering coefficient (C_p_), and small-world parameters (λ, γ, and σ) (51). For regional characteristics, we calculated the nodal global and local efficiency (52). The detailed definitions of these network measures can refer to (51) and Supplement 1.

#### Hub Distribution

To identify the hub distributions of WM networks in each group, we constructed the backbone network with consistent edges which exist in over 80% subjects for each group. Based on the backbone network, we identified the hub regions by sorting the nodal degree [K(i) > mean + std]. According to the categorization of the nodes into hub and non-hub regions, the edges were classified into rich-club, feeder and local connections (21, 22). Finally, the connection strength of each type of connections were calculated for each participant.

The graph analyses of brain networks were performed using the in-house software, GRETNA (http://www.nitrc.org/projects/gretna/) (53) and were visualized using BrainNet Viewer software (http://www.nitrc.org/projects/bnv/) (54).

### Statistical Analysis

#### Group Differences

Demographic factors and clinical scores including age, years of education, and neuropsychological scores among the three groups were compared using one-way analysis of variance (ANOVA). *Post-hoc* pairwise comparisons were then performed using *t*-tests. Gender distribution was compared with the Kruskal-Wallis one-way ANOVA. To determine the group difference in network metrics, comparisons were performed with univariate analysis of covariance (ANCOVA). *Post-hoc* pairwise comparisons were then performed using a general linear model. The effects of age, gender and years of education were adjusted for all of these analyses. For regional properties, multiple comparisons were corrected by using the false discovery rate (FDR) correction.

#### Network-Based Statistic (NBS)

To identify the specific connected components with significant different structural connections between each pair of groups, we used a NBS approach (55). Briefly, a primary cluster-defining threshold was first applied to identify connections above threshold, and the size (i.e., number of edges) of all connected components was determined. For each component, a corrected *p*-value was calculated using the null distribution of maximal connected component size, which was derived using the permutation approach (5,000 permutations). Notably, multiple linear regressions were performed to remove the effects of age, gender and years of education before the permutation tests. The detailed descriptions of the NBS analyses can refer to (55) and Supplement 1.

#### ROC Analysis

To determine the power of the connection strength of the NBS components to serve as potential biomarkers for clinical diagnosis of aMCI patients and differentiation between converters and non-converters, we performed a receiver operating characteristic (ROC) curve analysis for the strength of NBS components, which showed significant group differences.

#### Relationships Between Network Metrics and Clinical Scores

For the network metrics showing significant group differences, partial correlation analyses were performed between the network metrics and clinical scores for aMCI converters and non-converters separately, while removing the effects of age, gender and years of education. All the statistical analyses were performed using the MATLAB program (The MathWorks, Inc.).

### Support Vector Machine-Based Classification

To determine the discriminative ability of structural connectivity in separating aMCI patients from controls and separating converters from non-converters, we used the connection strength of the edges as the features for individual classification. For each pair of groups, we performed a support vector machine (SVM) classification, with a Gauss kernel function and the default settings of C = 1, coef = 0 and gamma as the reciprocal of the number of features in the LIVSVM Toolbox (http://www.csie.ntu.edu.tw/~cjlin/libsvm/) (56). Leave-one-out cross-validation (LOOCV) was used to evaluate the SVM model. Each subject was designated the test subject in turns while the remaining ones were used to train the SVM predictor. The hyperplane derived from the training subjects was then used to make a prediction about the group label of the test subject. Sensitivity, specificity, accuracy, and area under the curve (AUC) value were calculated to assess the performance of the classifier.

To avoid overfitting and reduce the redundant information, the F-score was calculated for each feature (connection), and the features with higher F-scores were used to train the model. The number of selected features (1%−20% with an interval of 1%) was decided by a grid search. The F-score was defined as (57):

(1) $$\begin{array}{r} \text{F}\left(\text{i}\right)=\frac{{\left({\overset{\bar{x}}{}}_{i}^{\left(+\right)}-{\overset{\bar{x}}{}}_{i}\right)}^{2}+{\left({\overset{\bar{x}}{}}_{i}^{\left(-\right)}-{\overset{\bar{x}}{}}_{i}\right)}^{2}}{\frac{1}{n_{+}-1}\sum_{k=1}^{n_{+}}{\left(x_{k,\;i}^{\left(+\right)}-{\overset{\bar{x}}{}}_{i}^{\left(+\right)}\right)}^{2}+\frac{1}{n_{-}-1}\sum_{k=1}^{n_{-}}{\left(x_{k,\;i}^{\left(-\right)}-{\overset{\bar{x}}{}}_{i}^{\left(-\right)}\right)}^{2}} \end{array}$$

where ${\overset{\bar{x}}{}}_{i},\;{\overset{\bar{x}}{}}_{i}^{\left(+\right)},\;{\overset{\bar{x}}{}}_{i}^{\left(-\right)}$ are the average of the *i*-th feature of the whole, positive, and negative data sets, respectively; $x_{k,\;i}^{\left(+\right)}$ is the *i*-th feature of the *k*-th positive instance; and $x_{k,\;i}^{\left(-\right)}$ is the *i*-th feature of the *k*-th negative instance.

The radial basis kernel function was defined as:

(2) $$\begin{array}{r} \text{K}\left(\text{x},\text{z}\right)=e^{\left(\frac{||x-z|{|}^{2}}{2\gamma^{2}}\right)} \end{array}$$

where x, z is the feature vector of a different instance; *e* is the Euler number, and γ is the hyper-parameter.

### Reproducibility Analysis

#### Effects of Different Thresholds

To test the stability of the results, we constructed individual WM networks with five different thresholds of fiber number (*w*_*ij*_ = 1, 2, 3, 4, 5). If the streamline number of an edge was less than the threshold, the edge weight was set to zero. For each threshold, the global network metrics were computed, and the group differences were assessed.

#### Effects of Different Parcellation Schemes

To evaluate the effects of different parcellation schemes on the network metrics, we further subdivided the low-resolution AAL (L-AAL) template into 1024 ROIs of equal size [i.e., high-resolution (H-1024)] (58). A high-resolution network was constructed for each participant and followed that with the same network analysis.

## Results

### Demographics and Neuropsychological Testing

No group differences were found in age, gender and years of education among the three groups. For clinical scores, aMCI patients showed a lower MMSE [*F*_(2, 75)_ = 20.29, *p* < 0.001], MoCA [*F*_(2, 75)_ = 58.54, *p* < 0.001], and AVLT scores [AVLT-immediate recall: *F*_(2, 75)_ = 53.07, *p* < 0.001; AVLT-delayed recall: *F*_(2, 75)_ = 59.08, *p* < 0.001; AVLT-recognition: *F*_(2, 75)_ = 20.22, *p* < 0.001] than controls. Between the two aMCI groups, lower MMSE and MoCA scores were observed in aMCI converters relative to non-converters (all *p* < 0.05; Table 1).

### Global Topology of the WM Structural Networks

**Figure 2:**
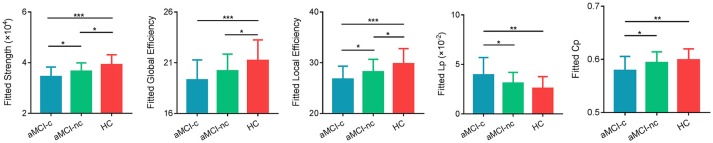
Global measures of the WM structural network were quantified in the aMCI converters, non-converters, and controls. The bars and error bars represent the fitted values and the standard deviations, respectively. The fitted values indicates the residuals of the original values of the network metrics after removing the effects of age, gender and years of education. The asterisk indicated a significant difference between groups. (^*^) represents a significant group difference at *p* < 0.05; (^**^) represents a significant group difference at *p* < 0.01; and (^***^) represents a significant group difference at p < 0.001.

Characteristic small-world organization of the WM networks (λ ≈ 1, γ > 1) were observed for both aMCI patients and control subjects. Among the three groups, ANCOVAs on the global network properties showed significant group effects in network strength [*F*_(2, 75)_ = 10.18, *p* = 0.0001], global efficiency [*F*_(2, 75)_ = 6.51, *p* = 0.0025], local efficiency [*F*_(2, 75)_ = 8.05, *p* = 0.0007], shortest path length [*F*_(2, 75)_ = 6.40, *p* = 0.0028] and clustering coefficient [*F*_(2, 75)_ = 5.20, *p* = 0.0078; Table 2] (Figure 2). In addition, *post-hoc* comparisons showed significantly reduced network strength, global efficiency and local efficiency in both aMCI converters and non-converters relative to the controls. Increased shortest path length and decreased clustering coefficient were found only in aMCI converters relative to controls. Between aMCI converters and non-converters, significant group differences were found in network strength [*t*_(47)_ = 2.28, *p* = 0.027], local efficiency [*t*_(47)_ = 2.19, *p* = 0.034), shortest path length [*t*_(47)_ = −2.12, *p* = 0.039], and clustering coefficient [*t*_(47)_ = 2.20, *p* = 0.033; Table 2; Figure 2].

**Table 2:** Group differences in global network metrics.

	**aMCI-c (*n* = 26)**	**aMCI-nc (*n* = 26)**	**HC (*n* = 26)**	***F* value**	***P* value**
Strength	220.4 ± 46.6	241.0 ± 37.3	268.6 ± 39.6	10.18	<0.001^a^^b^^c^
Global efficiency	12.1 ± 2.5	13.0 ± 1.9	14.1 ± 2.1	6.51	0.002^b^^c^
Local efficiency	18.3 ± 3.3	19.7 ± 2.6	21.4 ± 3.0	8.05	<0.001^a^^b^^c^
L_p_ (× 10^−2^)	8.66 ± 2.16	7.85 ± 1.24	7.22 ± 1.07	6.40	0.003^a^^b^
C_p_	0.53 ± 0.03	0.55 ± 0.02	0.55 ± 0.02	5.20	0.008^a^^b^
Lambda	1.18 ± 0.06	1.19 ± 0.03	1.19 ± 0.04	0.35	0.71
Gamma	3.21 ± 0.49	3.10 ± 0.30	3.03 ± 0.25	1.92	0.15
Sigma	2.72 ± 0.41	2.61 ± 0.24	2.55 ± 0.21	2.53	0.09

*Values are represented as the mean ± SD. Abbreviations: HC, healthy control; aMCI, amnestic mild cognitive impairment; aMCI-c, aMCI converters; aMCI-nc, aMCI non-converters. Lp, shortest path length; Cp, clustering coefficient*.

*The P-values were obtained with a univariate analysis of covariance (ANCOVA). Post-hoc pairwise comparisons were then performed using a general linear model. The effects of age, gender and years of education were adjusted for all of these analyses. P < 0.05 was considered significant*.

a *post-hoc paired comparisons showed a significant group difference between aMCI-c vs. aMCI-nc*.

b *post-hoc paired comparisons showed a significant group difference between aMCI-c vs. HC*.

c *post-hoc paired comparisons showed a significant group difference between aMCI-nc vs. HC*.

### Node-Based Analysis

**Figure 3:**
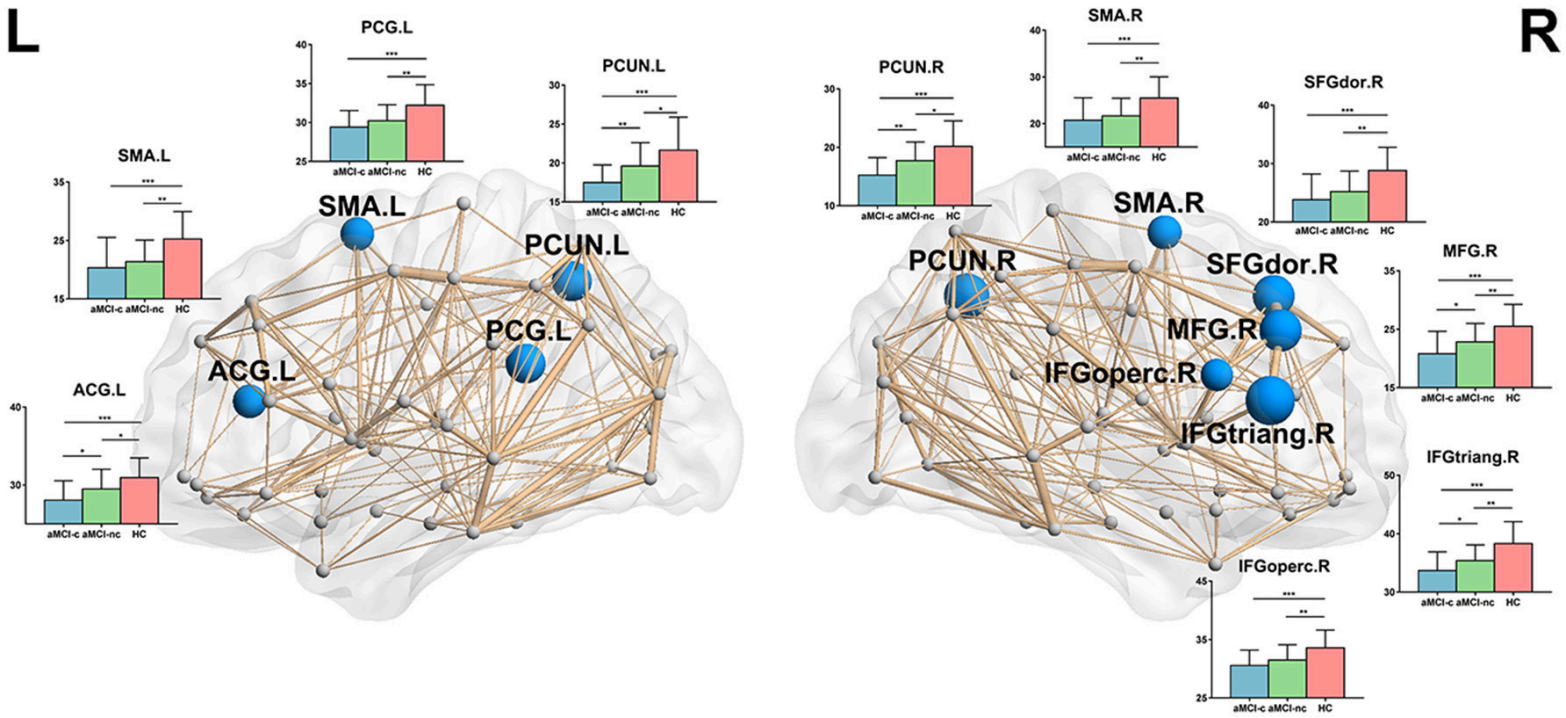
The distribution of brain regions with significant group effects in the nodal global efficiency among the three groups (*p* < 0.05, corrected). The node sizes indicate the significance of group differences in the nodal global efficiency. For each node, the bar and error bar represent the fitted values and the standard deviations, respectively, of the nodal global efficiency in each group. *post-hoc* tests revealed that all of these regions showed reduced nodal global efficiency in both aMCI converters and non-converters relative to controls. Several brain regions (5/10) showed more severe disruptions in aMCI converters compared with non-converters, including the bilateral precuneus, left anterior cingulate gyrus, right middle frontal gyrus, and right triangular part of the inferior frontal gyrus. (^*^) represents a significant group difference at *p* < 0.05; (^**^) represents a significant group difference at *p* < 0.01; and (^***^) represents a significant group difference at *p* < 0.001.

Following the discovery of a disrupted global network organization, we further localized the regions with altered nodal global and local efficiency. For nodal global efficiency, regions with significant group effects were mainly distributed in the frontal and parietal cortices, including 7 frontal regions (right dorsolateral superior frontal gyrus, right middle frontal gyrus, right opercular part of the inferior frontal gyrus, right triangular part of the inferior frontal gyrus, left anterior cingulate gyrus, bilateral supplementary motor area) and 3 parietal regions (left posterior cingulate gyrus, bilateral precuneus) (*p* < 0.05, corrected) (Figure 3). *Post-hoc* tests showed that all of these regions showed reduced global efficiency in both aMCI converters and non-converters relative to controls. In particular, several brain regions showed more severe disruptions in aMCI converters compared with non-converters, including the bilateral precuneus, left anterior cingulate gyrus, right middle frontal gyrus, and right triangular part of the inferior frontal gyrus (all *p* < 0.05).

**Figure 4:**
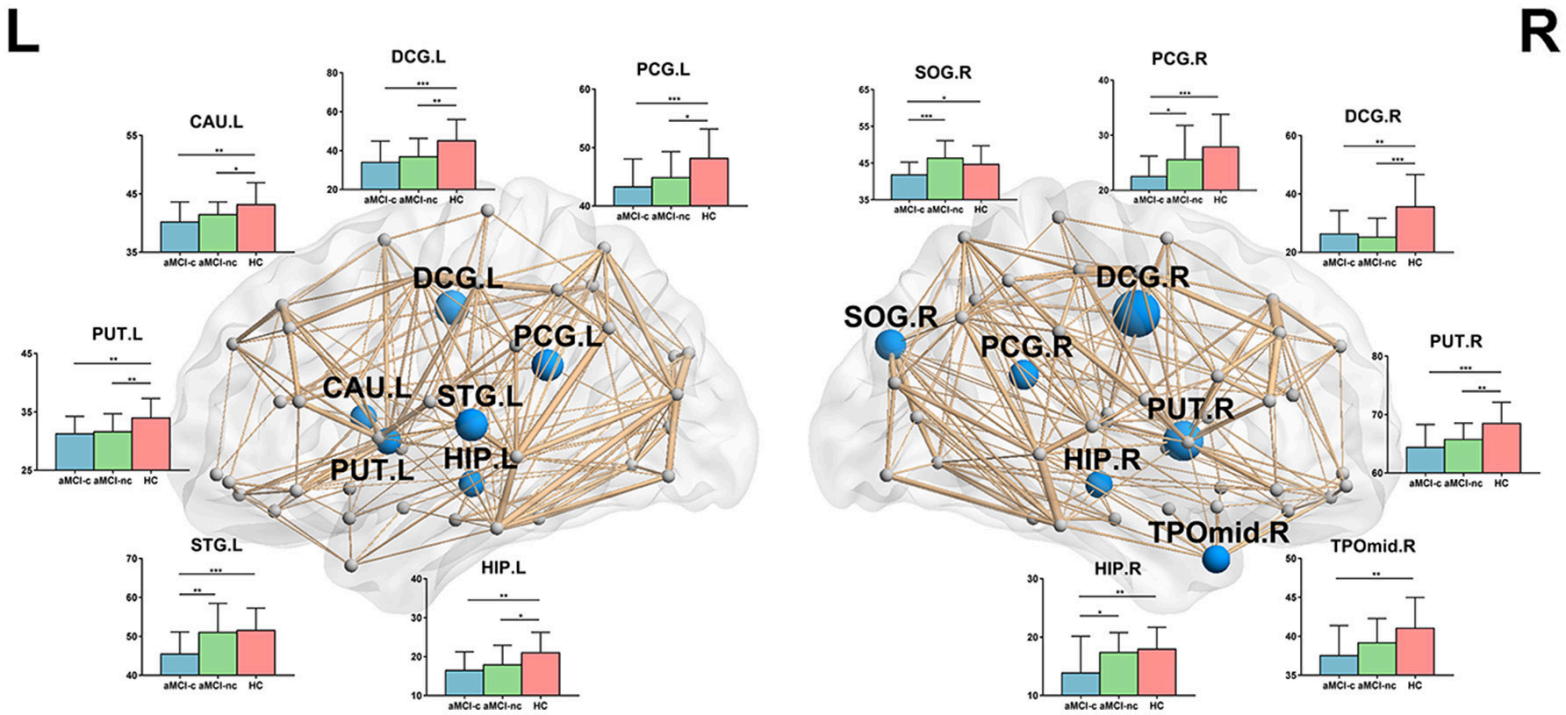
The distribution of brain regions with significant group effects in the nodal local efficiency among the three groups (*p* < 0.05, corrected). The node sizes indicate the significance of group differences in the nodal local efficiency. For each node, the bar and error bar represent the fitted values and the standard deviations, respectively, of the nodal local efficiency in each group. *post-hoc* tests revealed that all of these regions had a reduced nodal local efficiency in aMCI converters compared with controls. In seven of these regions, including the bilateral putamen, bilateral median cingulate, and paracingulate gyri, left posterior cingulate gyrus, left hippocampus and left caudate nucleus, reduced local efficiency was observed in aMCI non-converters compared with controls. Between the two aMCI groups, four regions (left superior temporal gyrus, right superior occipital gyrus, right posterior cingulate gyrus, and right hippocampus) showed a more severe disruption of local efficiency in the aMCI converters relative to non-converters. (^*^) represents a significant group difference at *p* < 0.05; (^**^) represents a significant group difference at *p* < 0.01; and (^***^) represents a significant group difference at *p* < 0.001.

For nodal local efficiency, regions with significant group effects were mainly distributed in the limbic cortices (bilateral median cingulate and paracingulate gyri and posterior cingulate gyrus), temporal cortices (left superior temporal gyrus, right temporal pole, and bilateral hippocampus), subcortical regions (left caudate nucleus and bilateral putamen) and right superior occipital gyrus (*p* < 0.05, corrected) (Figure 4). All of these regions had a reduced local efficiency in aMCI converters compared with controls. In seven of these regions, including the bilateral putamen, bilateral median cingulate and paracingulate gyri, left posterior cingulate gyrus, left hippocampus and left caudate nucleus, reduced local efficiency was observed in aMCI non-converters compared with controls. Between the two aMCI groups, four regions (left superior temporal gyrus, right superior occipital gyrus, right posterior cingulate gyrus, and right hippocampus) showed a more severe disruption of local efficiency in the aMCI converters relative to non-converters (all *p* < 0.05).

### Connectivity-Based Analysis

**Figure 5:**
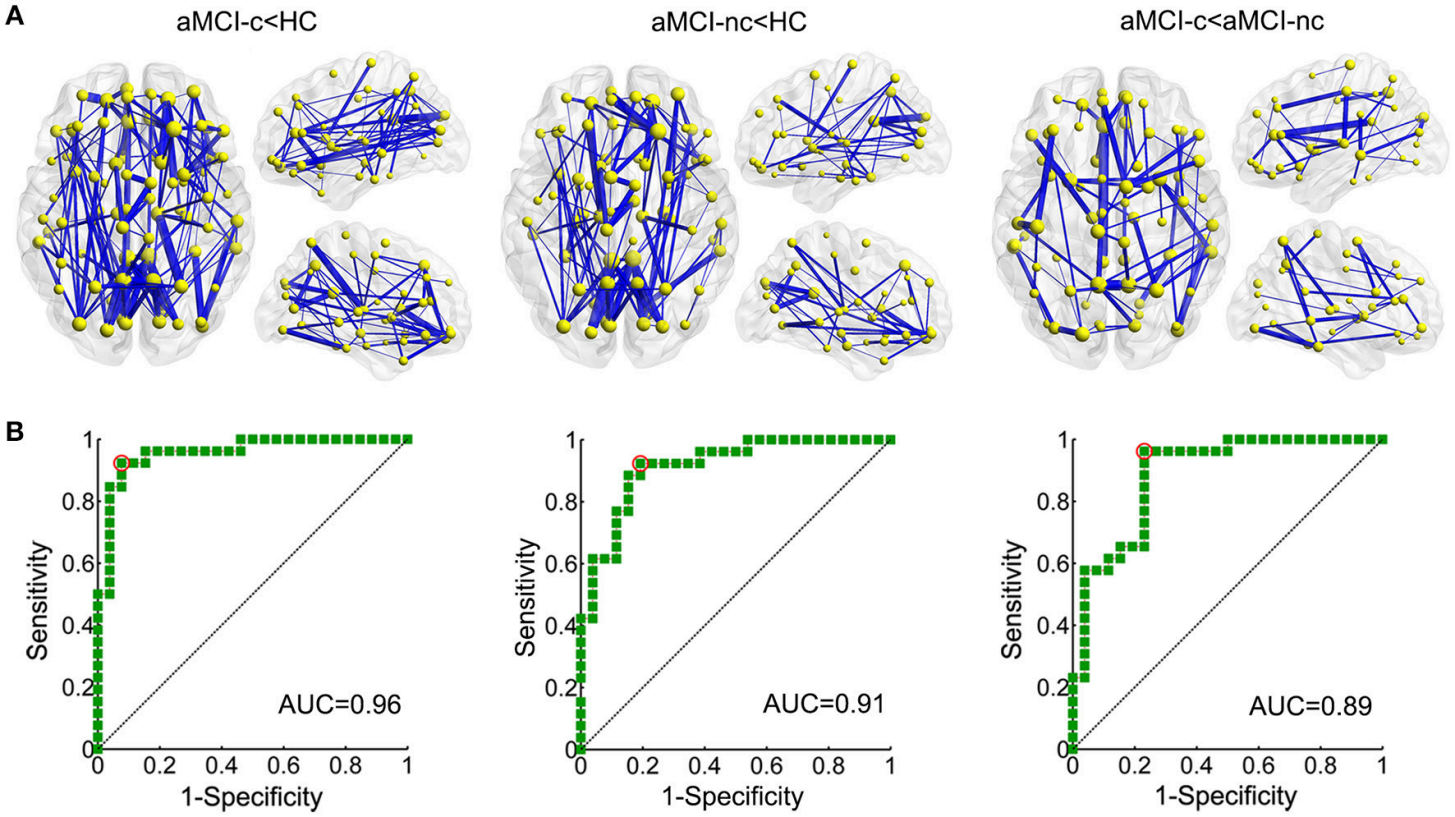
Altered structural connectivity between each pair of groups identified using NBS. **(A)** Compared to healthy controls, a single component with 83 nodes and 177 connections was altered in aMCI converters (*p* < 0.001, corrected) and a component with 73 nodes and 122 connections was detected in aMCI non-converters (*p* < 0.001, corrected). The comparison between aMCI converters and non-converters revealed a component with decreased strength in converters, which was composed of 70 nodes and 81 connections (*p* < 0.05, corrected). The edge sizes indicate the significance of the between-group differences. **(B)** ROC curve of the NBS component between aMCI converters and healthy controls (AUC = 0.96); between aMCI non-converters and healthy controls (AUC = 0.91); and between aMCI converters and non-converters (AUC = 0.89). (AUC, area under the curve).

NBS analyses were carried out to identify the disrupted connected components in patients. Compared to healthy controls, a single component with 83 nodes and 177 connections was altered in aMCI converters (*p* < 0.001, corrected) and a component with 73 nodes and 122 connections was detected in aMCI non-converters (*p* < 0.001, corrected) (Figure 5A). The involved regions had a widespread distribution across the frontal, temporal, parietal, occipital, and subcortical regions. The comparison between aMCI converters and non-converters revealed a component with decreased strength in converters, which was composed of 70 nodes and 81 connections (p < 0.05, corrected), mainly involving the bilateral precuneus, bilateral putamen, left anterior cingulate gyrus, right superior parietal gyrus, left middle temporal gyrus, left paracentral lobule, and left superior occipital gyrus (Figure 5A).

ROC analyses were performed to evaluate the discriminative ability of the disrupted component identified by NBS. The NBS component exhibited good performance for the discrimination between aMCI converters and healthy controls (with an AUC value of 0.96), between aMCI non-converters and healthy controls (with an AUC value of 0.91) and between aMCI converters and non-converters (with an AUC value of 0.89) (Figure 5B).

### Rich-Club Organization

**Figure 6:**
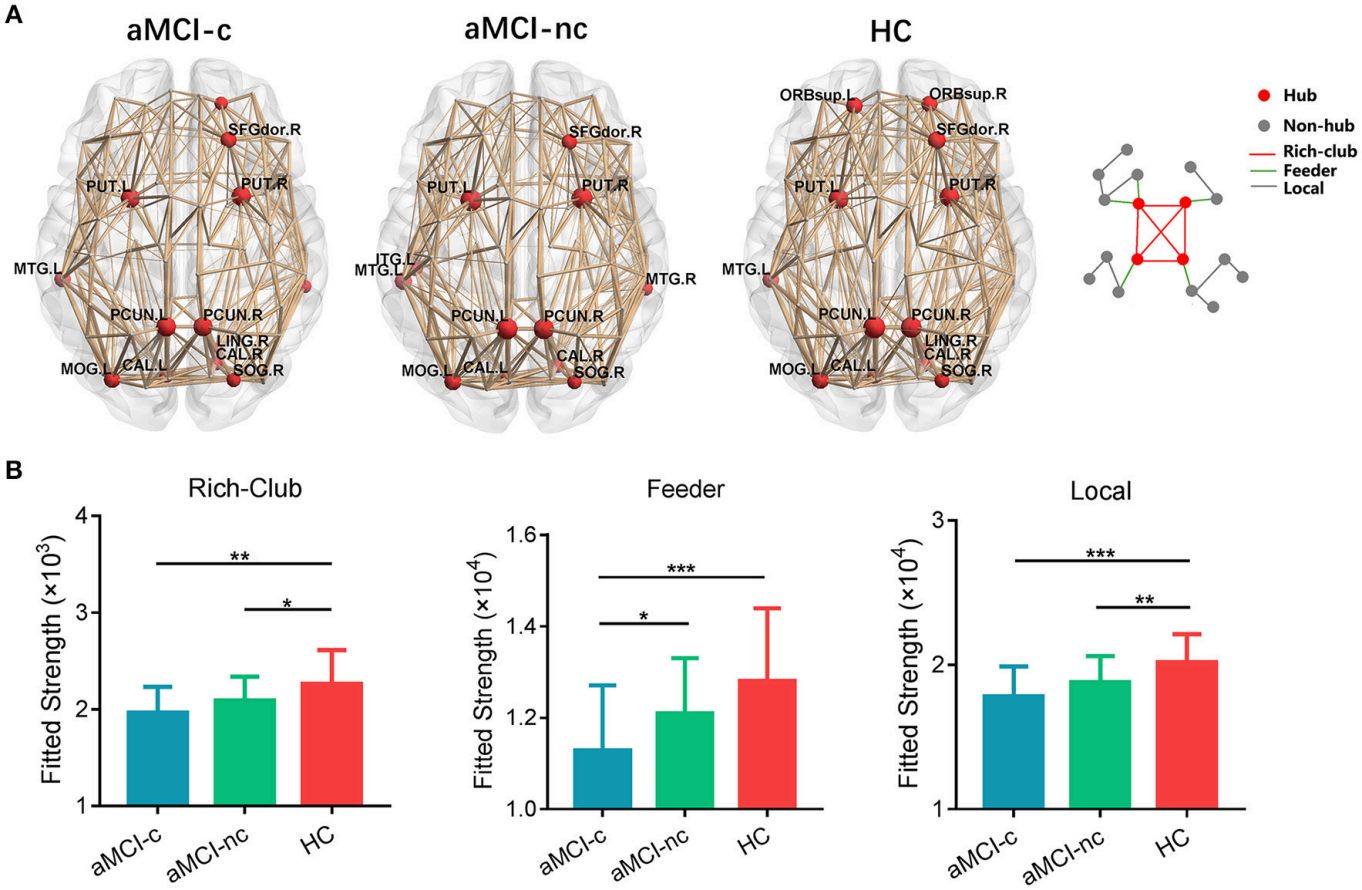
Hub distributions of the WM structural network in the aMCI converter, aMCI non-converter and control groups. **(A)** The hub nodes are shown in red with the node sizes indicating their nodal degree. The networks shown here were constructed by averaging the WM connection matrices of all the subjects in each group with a sparsity of 10%. **(B)** The group differences in the rich-club/feeder/local connection strengths. The bars and error bars represent the fitted values and the standard deviations, respectively, of the connection strength in each group. (^*^) represents a significant group difference at *p* < 0.05; (^**^) represents a significant group difference at *p* < 0.01; and (^***^) represents a significant group difference at *p* < 0.001.

Similar hub distributions were found across three groups (Figure 6A), mainly located in bilateral precuneus, bilateral putamen, right dorsolateral superior frontal gyrus, left middle temporal gyrus and several occipital regions. Several brain regions were identified as hubs only in the control group, such as bilateral orbital part of superior frontal gyrus. Among the three groups, significant group effects were identified in the strength of rich-club [*F*_(2, 75)_ = 6.67, *p* = 0.0022], feeder [*F*_(2, 75)_ = 7.25, *p* = 0.0013] and local [*F*_(2, 75)_ = 9.44, *p* = 0.0002] connections (Figure 6B). Compared with healthy controls, aMCI converters showed significant decreases in all three types of connections (all *p* < 0.005) and aMCI non-converters showed decreases in rich-club [*t*_(47)_ = 2.06, *p* = 0.045] and local [*t*_(47)_ = 2.82, *p* = 0.007] connections. Only feeder connections decreased significantly in aMCI converters compared with non-converters [*t*_(47)_ = 2.26, *p* = 0.028].

### Correlations Between Network Metrics and Neuropsychological Tests

**Figure 7:**
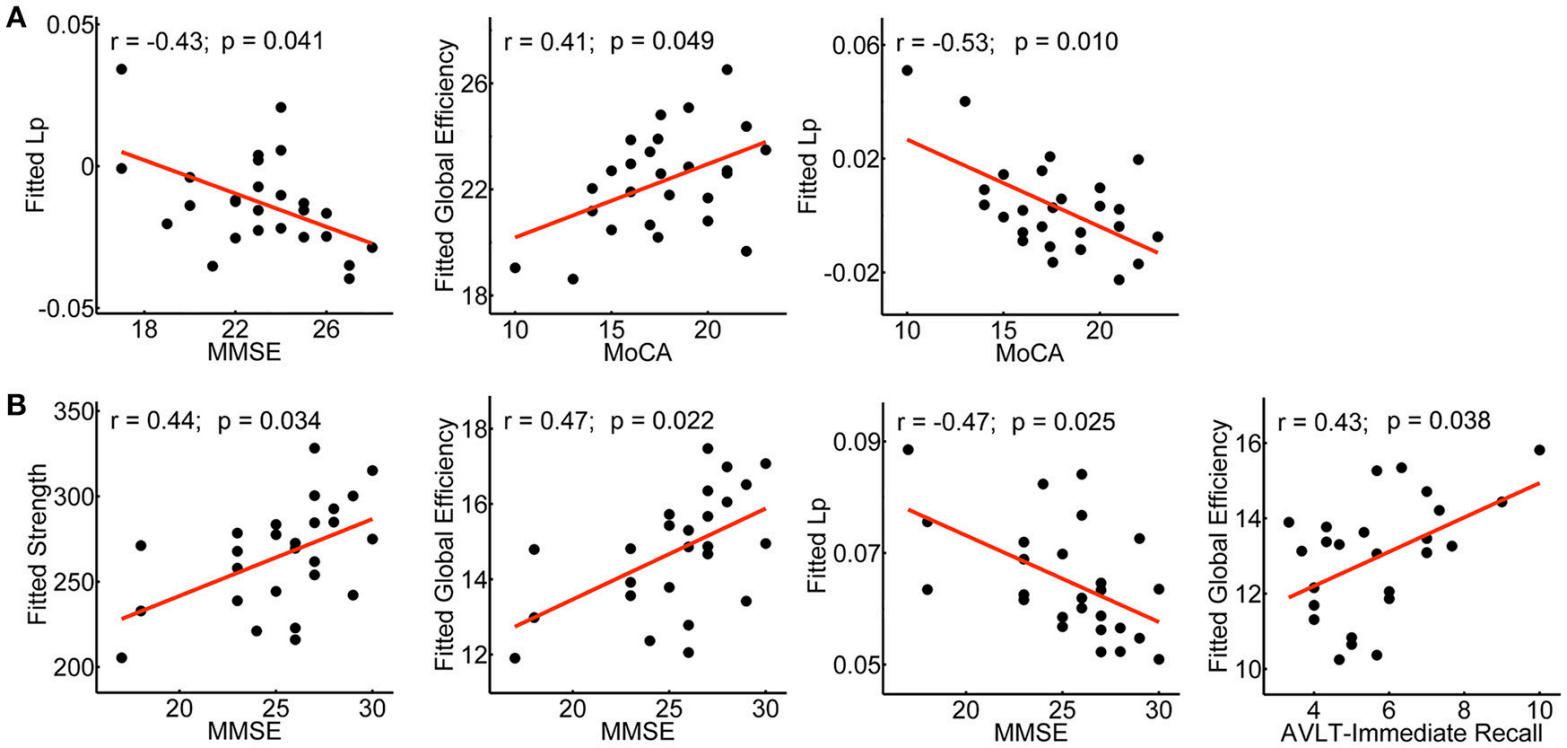
Clinical correlations of the network metrics in aMCI converters and non-converters. Plots showing the significant correlations between the network metrics and the clinical scores in aMCI converters **(A)** and non-converters **(B)**, respectively. The fitted values indicate the residuals of the original values of the network metrics after removing the effects of age, gender and years of education.

The relationship between network metrics and clinical scores were examined for aMCI converters and non-converters, respectively. In aMCI converters: MoCA was positively correlated with global efficiency (*r* = 0.41; *p* = 0.049), and negatively correlated with shortest path length (*r* = −0.53; *p* = 0.010); MMSE was negatively correlated with shortest path length (*r* = −0.43; *p* = 0.041) (Figure 7A). In aMCI non-converters: MMSE was positively correlated with network strength (*r* = 0.44; *p* = 0.034) and global efficiency (*r* = 0.47; *p* = 0.022), and negatively correlated with shortest path length (*r* = −0.47; *p* = 0.025); AVLT-Immediate Recall was positively correlated with global efficiency (*r* = 0.43; *p* = 0.038) (Figure 7B).

### Individual Classification of aMCI Converters and Non-converters

**Figure 8:**
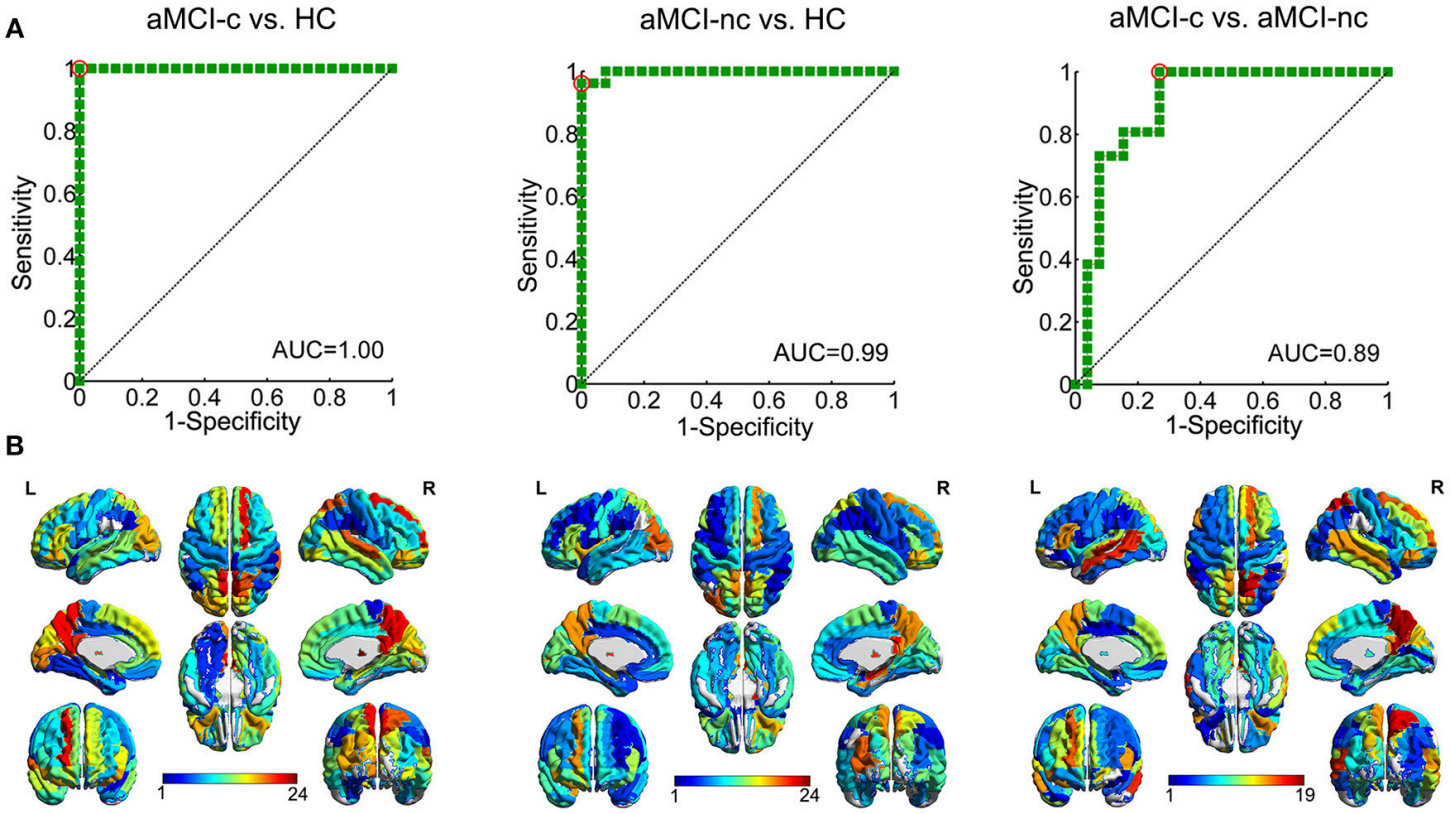
ROC curves of the SVM classification between each pair of groups. **(A)** ROC curve of the SVM classification between aMCI converters and healthy controls, AUC = 1.00; between aMCI non-converters and healthy controls, AUC = 0.99; and between aMCI converters and non-converters, AUC = 0.89. (AUC, area under the curve) **(B)** Regions to which the structural connections with the most discriminative power in the SVM classification was connected. The color shows the average number of edges selected as the features in each SVM classification connected to this region.

The results of SVM classification demonstrated good discriminative ability of structural connectivity in the differentiation between aMCI patients and controls and between aMCI converters and non-converters. The ROC curves for the classification between each pair of groups are shown in Figure 8A. For the discrimination between aMCI converters and controls, an AUC value of 1.00 was obtained, with an accuracy of 98.08%, sensitivity of 100% and specificity of 96.15%. Between aMCI non-converters and controls, an AUC value of 0.99 was obtained, with an accuracy of 98.08%, sensitivity of 100% and specificity of 96.15%. Between aMCI converters and non-converters, an AUC value of 0.89 was obtained, with an accuracy of 80.77%, sensitivity of 92.31%, and specificity of 69.23%. The effects of number of selected features on the classification accuracy were also evaluated (Figure S1).

The discriminative features for the classification were mapped onto the regions, which were rendered with the total number of connections from this region selected as features in the SVM classification (Figure 8B). For the classification between aMCI and controls, the most selected features were connections of the bilateral precuneus, bilateral posterior cingulate gyrus, right putamen, right thalamus, right dorsolateral superior frontal gyrus, left orbital part of the inferior frontal gyrus, and left caudate nucleus. For the classification between aMCI converters and non-converters, the most contributed features were connections of the bilateral precuneus, bilateral middle temporal gyrus, bilateral putamen, right medial superior frontal gyrus and left triangular part of the inferior frontal gyrus.

### Reproducibility of the Findings

#### Effects of Different Thresholds

For the different thresholds of network construction (w_ij_ = 1,2,3,4,5), similar group differences were found for network strength, global efficiency, local efficiency, and shortest path length (all *p* < 0.05) (Figure S2A).

#### Effects of Different Parcellation

For the high-resolution (H-1024) network analysis, significant group effects in network strength [*F*_(2, 75)_ = 10.14, *p* = 0.0001], global efficiency [*F*_(2, 75)_ = 9.40, *p* = 0.0002], local efficiency [*F*_(2, 75)_ = 6.41, *p* = 0.0027], and shortest path length [*F*_(2, 75)_ = 9.30, *p* = 0.0003] were observed (Figure S2B). *Post-hoc* analysis revealed significantly reduced network strength, global efficiency, local efficiency and increased shortest path length in both aMCI converters and non-converters relative to the controls (all *p* < 0.05). Between aMCI converters and non-converters, significant group differences were found in network strength [*t*_(47)_ = 2.06, *p* = 0.044], global efficiency [*t*_(47)_ = 2.06, *p* = 0.044], and shortest path length [*t*_(47)_ = −2.27, *p* = 0.028]. The group differences of global network metrics were comparable with those from low-resolution networks.

## Discussion

By combining DTI tractography and graph theoretical analyses, we demonstrated convergent and divergent topological alterations of the brain structural connectome in aMCI converters and non-converters. More severe disruptions of the structural connectome were identified in aMCI converters, especially in the DMN regions and connections. Importantly, the structural connectivity showed good discriminative ability in the differentiation of aMCI converters and non-converters, providing potential connectome-based markers for the early prediction of disease progression in aMCI patients.

### Global Network Disruption Between MCI Converters And Non-converters

First, we found similar patterns of global network alterations in both aMCI converters and non-converters. Compared with healthy controls, aMCI patients showed reduced network strength, global efficiency and local efficiency, but remained similar with respect to small-world parameters. These findings are consistent with our previous graph analysis of brain structural networks in aMCI patients (26, 27). As a disconnection disease, lower network global, and local efficiency were related to the widespread disruption of both long-range and short-range structural connectivity in aMCI patients, which indicated the pathological or degenerative alterations of WM in the early stage of AD. The possible mechanisms of structural disconnection may be due to cortical amyloid deposition, neural dysfunction, vascular damage, demyelination and so on (59–61).

Importantly, compared to aMCI non-converters, converters demonstrated lower network strength, local efficiency, and increased shortest path length even at baseline. Lower network strength was associated with sparse connectivity of brain networks, which indicated reduced WM integrity in the early phase of aMCI converters. This finding is in line with the evidence of more severe disruption of WM connectivity in MCI converters than non-converters with conventional DTI analyses (10, 12, 62). In addition, decreased local efficiency is mainly due to the loss of short-range connections among the neighborhood regions, and an increased shortest path length may be attributable to the disrupted long-range connections between remote regions, which is important for interregional effective integrity or prompt transfer of information in brain networks and constitutes the basis of cognitive processes (63). The alteration pattern of WM networks between converters and non-converters was similar to that in prior cross-sectional studies, which have identified network alterations with disease progression in AD and MCI patients (64–66). More severe disruptions of network properties in AD patients relative to MCI patients were found. Our study confirmed these cross-sectional reports of network dysfunction in AD and MCI and extended those with additional new findings.

Before disease transition, more severe structural or functional connectivity alterations already existed in the aMCI converters compared with non-converters (37–41). From the current study, we found that the network measures from DTI data are sensitive enough to detect the topological differences even at baseline, and correlated with the disease severity evaluated by clinical scores (MMSE, MoCA, and AVLT-Immediate Recall) in aMCI patients. Compared with the traditional regional or local brain measures, brain network studies provide a systematic perspective to investigate the disease progression and new insights into understanding the neuropathological mechanisms of disease conversion. Our results suggest the pivotal role of WM network disruption in the genesis of dementia and highlight the potential of a disease marker to identify patients at risk for dementia at an early stage.

### Regional/Connectivity Differences Between MCI Converters and Non-converters

Between aMCI patients and controls, significant differences in nodal global efficiency were mainly located in the bilateral precuneus, prefrontal cortex, and posterior cingulate gyrus, consistent with our previous network findings of aMCI patients (25–27). The reduced nodal global efficiency reflected a disrupted global integration of the structural connectivity in these regions, which may be due to more severe disconnection in AD-related hub brain regions concentrating most of the amyloid deposition (30, 31, 67–69). Furthermore, relative to aMCI non-converters, aMCI converters showed reduced nodal global efficiency in the bilateral precuneus, left anterior cingulate gyrus and right middle frontal gyrus, the regions that belong to the default mode network (DMN), which overlap with brain regions in distribution of early accumulation of cortical Aβ fibril (70), as well as to the pattern of hypometabolism found on FDG-PET studies (71) and of hypoperfusion on resting MR perfusion studies of AD patients (72). A functional MRI study has suggested the significant predictive value of DMN connectivity in predicting the disease progression to AD in MCI patients (73). Amyloid accumulation started from the DMN and was correlated with hypoconnectivity of the DMN (70). The association between amyloid accumulation and cognition was found to be influenced by functional connectivity of the DMN (74). Moreover, a prior DTI study has suggested that an increased amyloid burden is related to changes in topology of WM network architecture in MCI and AD patients (60), suggesting that pathological propagation affects large-scale functional and structural brain networks with disease progression. Notably, the most significant differences between converters and non-converters were located in the bilateral precuneus; as one of the most important regions of the DMN, the precuneus plays a critical role in memory processing and AD progression. A previous structural MRI-based network study has found that betweenness centrality of the precuneus is associated with cognitive decline (75), which may suggest a key role of the precuneus in the disease conversion of aMCI patients.

Meanwhile, group differences in nodal local efficiency were mainly located in the bilateral hippocampus, middle and posterior cingulate gyri, superior and middle temporal gyri, which were characteristic AD-signature regions (9). Previous neuroimaging studies have also reported the structural or functional alterations in these brain regions in AD and MCI patients (27, 76–78). Reduced local efficiency of these regions may reflect local impairment in functional segregation of episodic memory, which may be related to the structural disruptions of short-range connections within the memory network which centered on the hippocampus (79–81). Relative to non-converter, reduced nodal local efficiency in the left superior temporal gyrus, right hippocampus and right superior occipital gyrus were found in aMCI converters. These regions were also identified as features for predicting progression to AD in MCI patients based on amyloid-PET (82).

Similar hub distributions were found across three groups, which were consistent with previous findings (27, 83). Hubs play a pivotal role in global information transfer and seem to be vulnerable and preferentially affected in AD patients (29). In our study, both hub and non-hub regions showed decreased efficiency and all categories of edges showed lower strength in aMCI patients. Between aMCI converter and non-converters, only feeder connections showed progressive disruption. We speculate that aMCI initiates with a widespread disruption of WM connectivity, and alterations in feeder connections may be with important predictive value for the disease progression.

Machine learning approaches for the individual prediction of disease progression Identifying sensitive and early biomarkers for the individual prediction of disease progression is important for early disease diagnosis and precise medicine. Machine learning approaches with big multimodality data provide a promising area for future intelligent computer-aided-diagnosis (84). For AD and MCI, a number of previous studies have tested different imaging, CSF or neuropsychological measures for the early prediction of disease conversion (9, 85–87). Based on the brain structure connectome and SVM classification, we obtained a high classification accuracy of 98% between aMCI patients and controls. Even between converters and non-converters, the accuracy can reach 81%, which is comparable and even higher than previous results (10, 12, 14, 38, 42, 88). This finding suggested the potential utility of brain structural connectivity/connectome-based markers for the individual prediction of disease conversion, which may provide biologically relevant information not present in other imaging markers.

### Methodological Issues

Several methodological issues should be addressed. First, the results were limited by the small sample size. In the future studies, several large publicly available datasets, such as ADNI, should be used as an independent cohort for validating the reproducibility of our findings. Second, we only identified abnormalities in patients with aMCI converters and non-converters at baseline, and longitudinal follow-up studies of the same study population are needed to verify the effects of early imaging markers for disease prediction. Third, we only studied WM structural networks. In future studies, the combination of the multimodal imaging and conventional pathological biomarkers would contribute to a more comprehensive prediction of the progression from aMCI to AD dementia. Finally, some newly developed network analysis approaches, such as multiplex networks, can help early AD classification (33). These approaches deserve a further investigation in future studies.

## Conclusions

By using DTI tractography combined with graph analysis, our study demonstrated more severe disrupted topological organization of brain structural connectome in aMCI converters compared with non-converters, providing potential connectivity/connectome-based biomarkers for the early prediction of disease progression in aMCI patients.

## Author Contributions

YS, QB, and XW: manuscript preparation and drafting. YS, XW, YH, and JL: clinical assessments and data acquisition. PC and YH: clinical diagnosis. QB, XH, HL, XL, TM, and NS: data analysis and interpretation. NS and YH: study conception and design. YS, QB, and XW contributed equally to this work. All authors have contributed to the manuscript revising and editing critically for important intellectual content and given final approval of the version, agreed to be accountable for all aspects of the work in ensuring that questions related to the accuracy or integrity of any part of the work are appropriately investigated and resolved.

### Conflict of Interest Statement

The authors declare that the research was conducted in the absence of any commercial or financial relationships that could be construed as a potential conflict of interest.
