# Supplementary material for: Prediction of Conversion From Amnestic Mild Cognitive Impairment to Alzheimer's Disease Based on the Brain Structural Connectome
Source: Front Neurol. 2019 Jan 10;9:1178. doi: 10.3389/fneur.2018.01178 (PMC6335339; doi:10.3389/fneur.2018.01178)
Supplement: Supplementary file 1 [file Table_1.DOC]

**Supplement 1**

**Text S1. Definitions of network metrics**

For the global network metrics, we quantified the network strength (S_p_), global efficiency (E_glob_), local efficiency (E_loc_), shortest path length (L_p_), clustering coefficient (C_p_) and small-world parameters (λ, γ and σ) . For the regional characteristics, we considered the nodal global and local efficiency . All network analyses were performed using the GRETNA software (http://www.nitrc.org/projects/gretna/). Detailed definitions of the network metrics are provided as follows.

*Network strength.* For a network (graph) G with N nodes and K edges, we calculated the strength of G as:

$$S_{p}(G)=\frac{1}{N}\sum_{i\in G} S(i)$$

where S(i) is the sum of the edge weights w_ij_ linking to node i. The strength of a network is the average of the strength across all of the nodes in the network.

*Small-world properties.* Small-world network parameters (clustering coefficient, C_p_ and shortest path length, L_p_) were originally proposed by Watts and Strogatz . In this study, we investigated the small-world properties of the weighted brain networks.

The clustering coefficient of a node i, C(i), which was defined as the likelihood of whether the neighborhoods were connected with each other or not, was computed as follows :

$$C(i)=\frac{2}{k_{i}(k_{i}-1)}\sum_{j,k} (\bar{w}_{ij}\bar{w}_{jk}\bar{w}_{ki})^{1/3}$$

where k_i_ is the degree of node i and $\bar{w}$ is the weight of edge, which is scaled by the largest weight of the network. The clustering coefficient is zero if the nodes are isolated or have just one connection, i.e., k_i_ = 0 or k_i_ = 1. The clustering coefficient, C_p_, of a network is the average of the clustering coefficient over all nodes and indicates the extent of the local interconnectivity or cliquishness in a network .

The path length between any pair of nodes (e.g., node i and node j) is defined as the sum of the edge lengths along this path. For weighted networks, the length of each edge was assigned by computing the reciprocal of the edge weight, 1/w_ij_. The shortest path length, L_ij_, is defined as the length of the path for node i and node j with the shortest length. The shortest path length of a network was computed as follows:

$$L_{p}(G)=\frac{1}{N(N-1)}\sum_{i\neq j\in G} L_{ij}$$

where N is the number of nodes in the network. The L_p_ of a network quantifies the ability for information to propagate in parallel.

To examine the small-world properties, the clustering coefficient, C_p_, and the shortest path length, L_p_, of the brain networks were compared with those of random networks. In this study, we generated 100 matched random networks, which had the same number of nodes, edges, and degree distribution as the real networks . Of note, we retained the weight of each edge during the randomization procedure such that the weight distribution of the network was preserved. Furthermore, we computed the normalized L_p_, $\lambda=L_{p}^{real}/L_{p}^{rand}$ and the normalized C_p_,$\gamma=C_{p}^{real}/C_{p}^{rand}$，where $L_{p}^{rand}$ and $C_{p}^{rand}$ are the mean L_p_ and the mean C_p_ of 100 matched random networks, respectively. Importantly, two parameters correct the differences in the edge number and degree distribution of the networks across individuals. A real network would be considered small-world if $\gamma>1$ and $\lambda\approx1$ . Thus, a small-world network not only has a higher local interconnectivity, but it also has an approximately equivalent shortest path length compared with random networks. These two measurements can be summarized into a simple quantitative metric, small-worldness,$\sigma=\gamma/\lambda$, which is typically greater than 1 for small-world networks .

*Network efficiency*. The global efficiency of G measures the global efficiency of the parallel information transfer in the network , which can be computed as:

$$E_{glob}(G)=\frac{1}{N(N-1)}\sum_{i\neq j\in G} \frac{1}{L_{ij}}$$

where L_ij_ is the shortest path length between node i and node j in G.

The local efficiency of G reveals how much the network is fault tolerant and shows how efficient the communication is among the first neighbors of the node i when it is removed. The local efficiency of a graph is defined as:

$$E_{loc}(G)=\frac{1}{N}\sum_{i\in G} E_{glob}(G_{i})$$

where G_i_ denotes the subgraph composed of the nearest neighbors of node i.

*Regional nodal characteristics.* To determine the nodal (regional) characteristics of the brain networks, we computed the nodal global and nodal local efficiency.

The nodal global efficiency, nE_glob_(i) is defined as :

$$nE_{glob}(i)=\frac{1}{N-1}\sum_{i\neq j\in G} \frac{1}{L_{ij}}$$

where L_ij_ is the shortest path length between node i and node j in G. The nodal global efficiency measures the average shortest path length between a given node i and all of the other nodes in the network.

The nodal local efficiency, nE_loc_(i) is defined as :

$$nE_{loc}(i)=E_{glob}(G_{i})$$

where G_i_ denotes the subgraph composed of the nearest neighbors of node i.

**Text S2. Network-based statistic**

To localize the specific connected subnetwork (component) with the different structural connections between each pair of groups, we used a network-based statistic (NBS) approach . First, the significant nonzero connections within each group were detected by performing a nonparametric one-tailed sign test. For each pair of brain regions, the sign test was performed with the null hypothesis that there is no existing connection, that is, “fiber number = 0.” The Bonferroni method was then used to correct for multiple comparisons (i.e., 90*89/2 = 4005 pairs of regions) at *p* = 0.05. Next, the nonzero connections within either the patient or control groups were detected and combined into a connection mask. The NBS approach was then conducted within the connection mask, where a primary threshold (*p* = 0.05) was first applied to a *t* statistic (two-sample one-tailed *t* tests). This *t* statistic was computed for each link to define a set of suprathreshold links among which any connected components and their size (number of links) could then be determined. To estimate the significance for each component, the null distribution of the connected component size was empirically derived using a nonparametric permutation approach (10,000 permutations). For each permutation, all of the subjects were randomly reallocated into two groups, and the *t* statistic was computed independently for each link. Next, the threshold (*p* = 0.05) was used to generate suprathreshold links among which the maximal connected component size was recorded. Finally, for a connected component of size *M* that was found in the right grouping of controls and patients, the corrected *p* value was determined by calculating the proportion of the 10,000 permutations for which the maximal connected component was larger than *M*. Of note, the effects of age, gender, and years of education were removed by a regression analysis performed before the statistical analysis of connections. For a detailed description, see the study by .

**References**

Achard, S., Bullmore, E., 2007. Efficiency and cost of economical brain functional networks. PLoS Comput Biol 3, e17.

Humphries, M.D., Gurney, K., 2008. Network 'small-world-ness': a quantitative method for determining canonical network equivalence. PLoS One 3, e0002051.

Latora, V., Marchiori, M., 2001. Efficient behavior of small-world networks. Phys Rev Lett 87, 198701.

Maslov, S., Sneppen, K., 2002. Specificity and stability in topology of protein networks. Science 296, 910-913.

Onnela, J.P., Saramaki, J., Kertesz, J., Kaski, K., 2005. Intensity and coherence of motifs in weighted complex networks. Phys Rev E Stat Nonlin Soft Matter Phys 71, 065103.

Rubinov, M., Sporns, O., 2010. Complex network measures of brain connectivity: uses and interpretations. Neuroimage 52, 1059-1069.

Watts, D.J., Strogatz, S.H., 1998. Collective dynamics of 'small-world' networks. Nature 393, 440-442.

Zalesky, A., Fornito, A., Bullmore, E.T., 2010. Network-based statistic: identifying differences in brain networks. Neuroimage 53, 1197-1207.

### Table S1. Cortical and subcortical regions of interest defined in the study

| **Index** | **Regions** | **Abbr.** | **Index** | **Regions** | **Abbr.** |
| --- | --- | --- | --- | --- | --- |
| (1,2) | Precental gyrus | PreCG | (47,48) | Lingual gyrus | LING |
| (3,4) | Superior frontal gyrus, dorsolateral | SFGdor | (49,50) | Superior occipital gyrus | SOG |
| (5,6) | Superior frontal gyrus, orbital part | ORBsup | (51,52) | Middle occipital gyrus | MOG |
| (7,8) | Middle frontal gyrus | MFG | (53,54) | Inferior occipital gyrus | IOG |
| (9, 10) | Middle frontal gyrus, orbital part | ORBmid | (55,56) | Fusiform gyrus | FFG |
| (11,12) | Inferior frontal gyrus, opercular part | IFGoperc | (57,58) | Postcentral gyrus | PoCG |
| (13,14) | Inferior frontal gyrus, triangular part | IFGtriang | (59,60) | Superior parietal gyrus | SPG |
| (15,16) | Inferior frontal gyrus, orbital part | ORBinf | (61,62) | Inferior parietal, but supramarginal and angular gyri | IPL |
| (17,18) | Rolandic operculum | ROL | (63,64) | Supramarginal gyrus | SMG |
| (19,20) | Supplementary motor area | SMA | (65,66) | Angular gyrus | ANG |
| (21,22) | Olfactory cortex | OLF | (67,68) | Precuneus | PCUN |
| (23,24) | Superior frontal gyrus, medial | SFGmed | (69,70) | Paracentral lobule | PCL |
| (25,26) | Superior frontal gyrus, medial orbital | ORBsupmed | (71,72) | Caudate nucleus | CAU |
| (27,28) | Gyrus rectus | REC | (73,74) | Lenticular nucleus, putamen | PUT |
| (29,30) | Insula | INS | (75,76) | Lenticular nucleus, pallidum | PAL |
| (31,32) | Anterior cingulate and paracingulate gyri | ACG | (77,78) | Thalamus | THA |
| (33,34) | Median cingulate and paracingulate gyri | DCG | (79,80) | Heschl gyrus | HES |
| (35,36) | Posterior cingulate gyrus | PCG | (81,82) | Superior temporal gyrus | STG |
| (37,38) | Hippocampus | HIP | (83,84) | Temporal pole: superior temporal gyrus | TPOsup |
| (39,40) | Parahippocampal gyrus | PHG | (85,86) | Middle temporal gyrus | MTG |
| (41,42) | Amygdala | AMYG | (87,88) | Temporal pole: middle temporal gyrus | TPOmid |
| (43,44) | Calcarine fissure and surrounding cortex | CAL | (89,90) | Inferior temporal gyrus | ITG |
| (45,46) | Cuneus | CUN |  |  |  |

Note: The regions are listed in terms of a prior template of an AAL-atlas (Tzourio-Mazoyer et al., 2002).


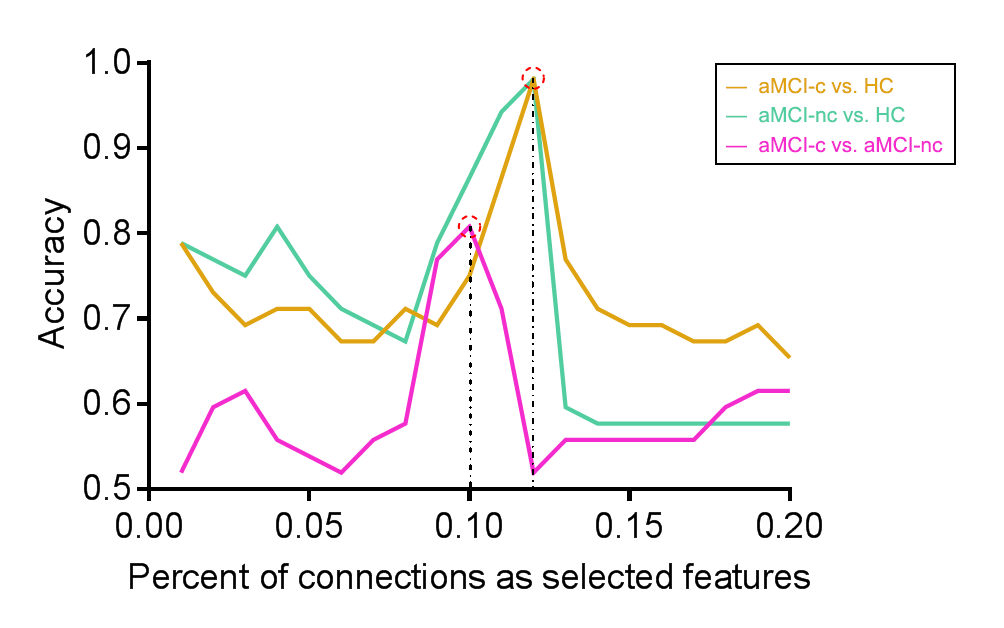


**Figure S1.** Evaluation of the effects of selected feature number on the classification accuracy. For each pair of groups, the classification accuracy was calculated under each threshold with different number of selected features (1% - 20% with an interval of 1%). For the classification between aMCI converters/non-converters and controls, the highest accuracy was obtained at the threshold with 12% of connections as selected features. For the classification between aMCI converters and non-converters, the highest accuracy was obtained at the threshold with 10% of connections as selected features.


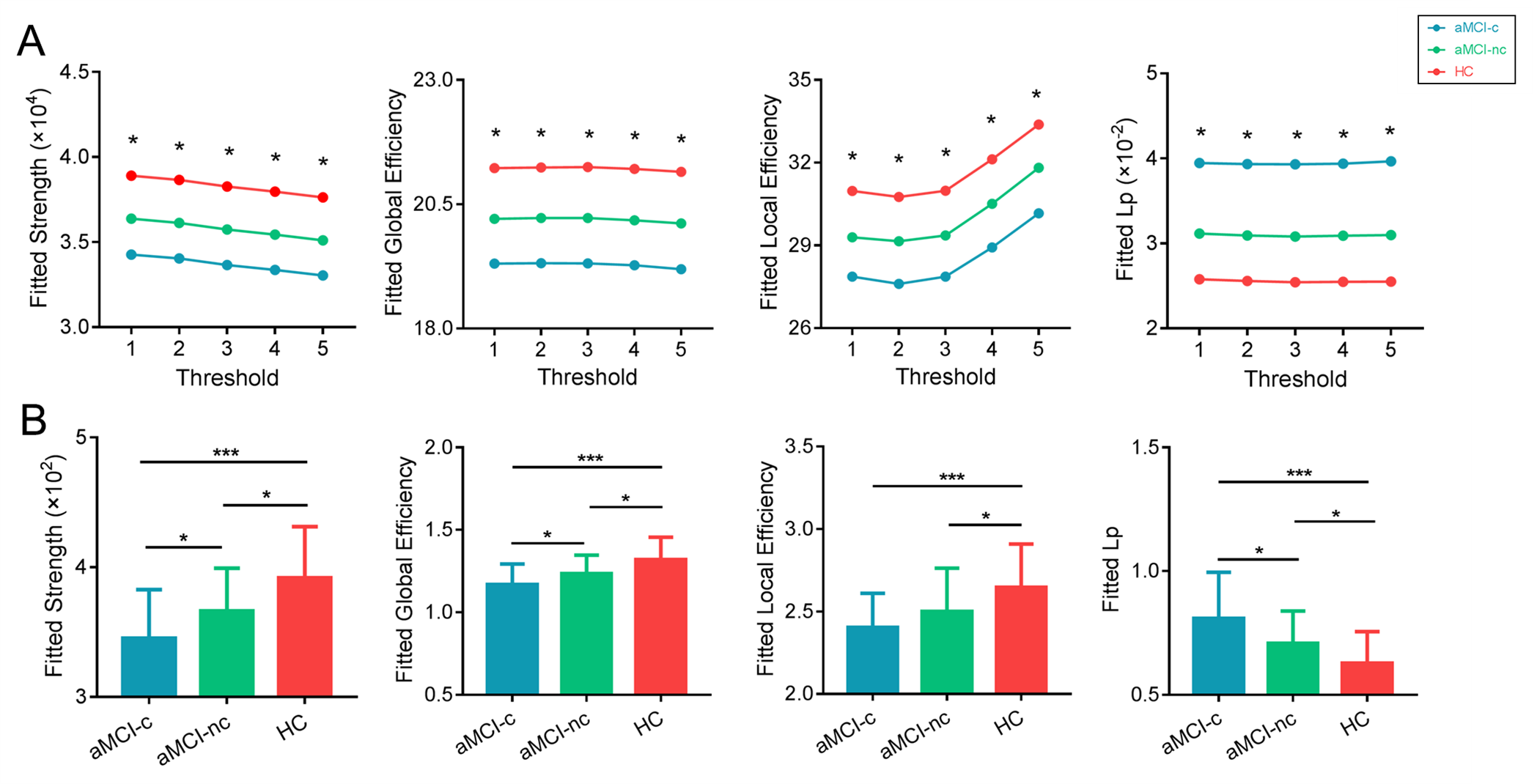


**Figure S2.** The reproducibility of the network analysis. (A) The group comparisons of the global network measures under 5 different thresholds (w_ij_ = 1, 2, 3, 4, 5) were performed using a general linear regression model with age, gender and years of education as covariates. The data points marked with a star indicate a significant group difference (p < 0.05) in the network metric under the threshold. (B) The group differences of the global network metrics of a high-resolution (H-1024) network were quantified between the groups. The bars and error bars represent the fitted values and the standard deviations, respectively. The fitted values indicate the residuals of the original values of the network metrics after removing the effects of age, gender and years of education. (*) represents a significant group difference at p < 0.05; (**) represents a significant group difference at p < 0.01; and (***) represents a significant group difference at p < 0.001.
