# Supplementary figures and images for: Prediction of Conversion From Amnestic Mild Cognitive Impairment to Alzheimer's Disease Based on the Brain Structural Connectome
Source: Front Neurol. 2019 Jan 10;9:1178. doi: 10.3389/fneur.2018.01178 (PMC6335339; doi:10.3389/fneur.2018.01178)

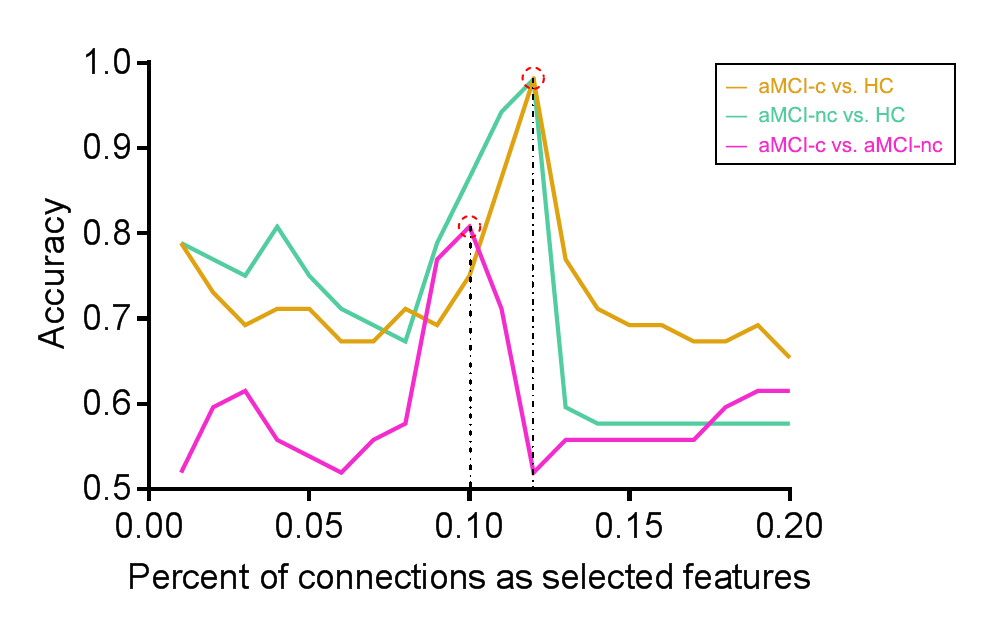

Supplement: Supplementary file 2 [file Image_1.TIF]

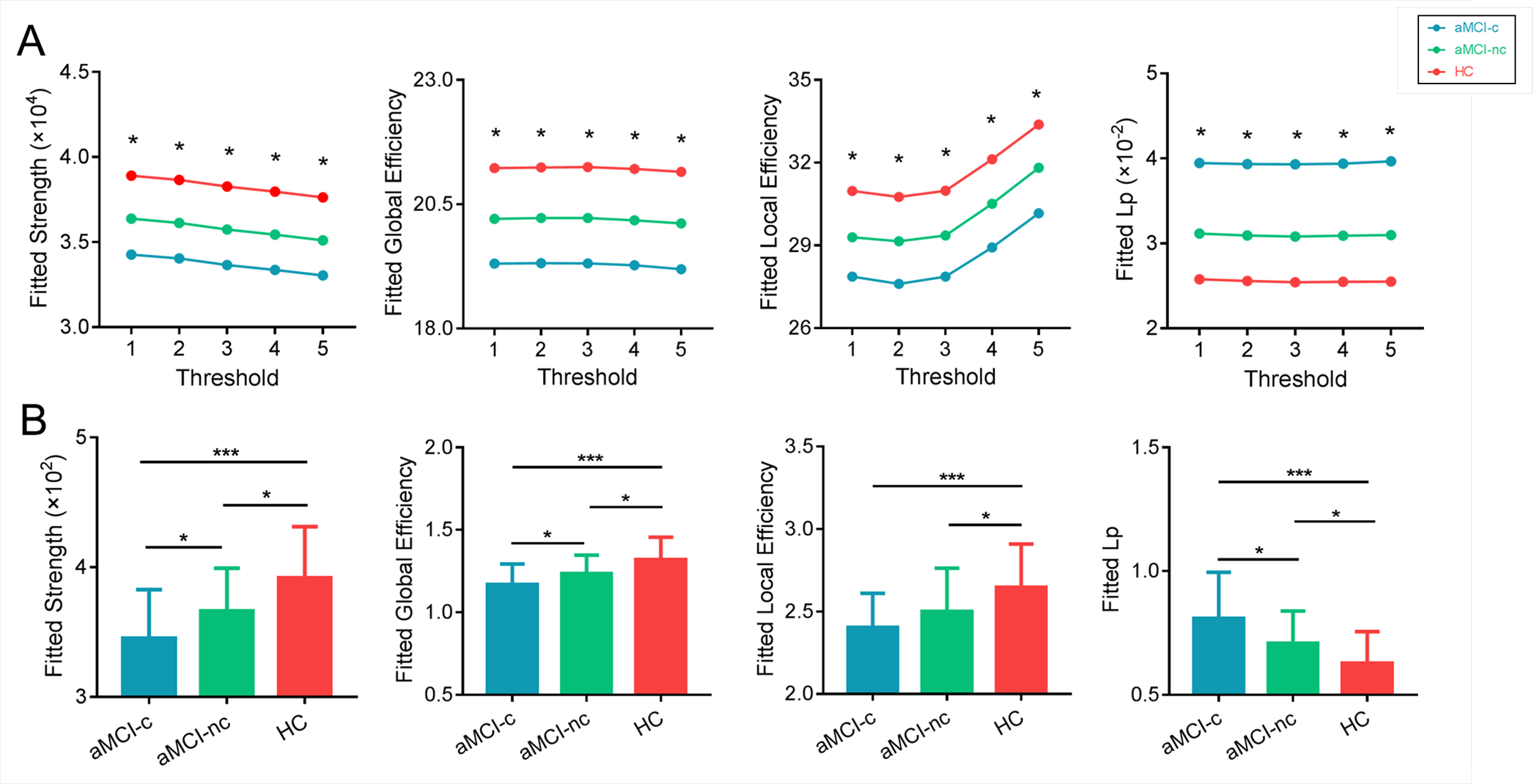

Supplement: Supplementary file 3 [file Image_2.TIF]
